# An exploration of industry expert perception of Canadian equine welfare using a modified Delphi technique

**DOI:** 10.1371/journal.pone.0201363

**Published:** 2018-07-30

**Authors:** Cordelie DuBois, Helen Hambly Odame, Derek B. Haley, Katrina Merkies

**Affiliations:** 1 Department of Animal Biosciences, University of Guelph, Guelph, Ontario, Canada; 2 Campbell Centre for the Study of Animal Welfare, University of Guelph, Guelph, Ontario, Canada; 3 School of Environmental Design and Rural Development, University of Guelph, Guelph, Ontario, Canada; 4 Department of Population Medicine, Ontario Veterinary College, University of Guelph, Guelph, Ontario, Canada; Universidade do Porto Instituto de Biologia Molecular e Celular, PORTUGAL

## Abstract

The diversity of sectors that comprise the equine industry makes reaching a consensus regarding welfare issues a challenge. To allow for productive discussion, equine professionals (n = 34) chosen to represent the diverse specializations from across Canada were surveyed using the Delphi technique—a survey technique employing multiple, iterative “rounds” to consolidate viewpoints—to gather and consolidate information regarding areas of welfare concern in the Canadian equine industry. Only participants who completed the prior round could participate in subsequent rounds. In the first round, respondents were asked to identify examples of welfare issues. Qualitative analysis was used to sort and group answers based on their similarities. Participants identified 12 welfare issues best addressed at the individual horse level, and an additional 12 welfare issues best addressed at the industry level. In the second (n = 24) and third (n = 14) rounds, welfare issues, solutions, and potential motives were consolidated based on order ranking. Themes of “ignorance” and “lack of knowledge” identified throughout all three rounds were cited as both potential risks to welfare as well as motives leading to poor welfare situations. Responses in this study suggest that in order to improve the welfare of equids in the Canadian industry, equine professionals propose that a greater effort is required to help educate industry members and stakeholders such that, through daily routine care and management, higher standards of welfare can be attained.

## Introduction

In Canada, the equine industry is a diverse and wide-spread industry composed of a variety of disciplines and uses of the horse. Different management styles and ways of viewing the horse (as a companion as opposed to a commodity, for example) further contribute to the industry’s range of both participants and viewpoints. It is therefore difficult to define “the equine industry”, and this in itself presents its own challenges with respect to research, particularly regarding welfare. With no standard method of managing horses, standardized research results are not applicable across the board, and determining the level of welfare of animals housed in a variety of ways becomes problematic.

With the recent revision of the National Farm Animal Care Council’s Code of Practice for the Care and Handling of Equines (NFACC) [1], it has become important not only to understand the current state of equine welfare in Canada but also to understand the perception of the industry by those active in it. Although the 2010 Canadian Horse Industry Profile Survey [2] provided new insight into the demographics of the equine industry, it did not address the industry’s perception of itself or of the welfare status of its horses. In this area there is a marked deficit in published data, limiting the effectiveness of efforts to understand or improve the industry as a whole. How welfare is perceived can have a significant impact on people’s willingness to change existing practices, especially when these changes might result in increased costs [3]. Identifying areas of concern within the industry through dialogue with participants has the benefit of directing future research towards a better understanding and the development of a process aimed at resolving these issues. This is made challenging by the diversity of equine industry participants.

A survey method called the Delphi technique (created originally by the Rand Corporation [4]) has been used to gain insight into the perceptions of a group of experts in a given topic, as well as to reach a consensus regarding subject matter about which people have diverse opinions [5]. Using multiple rounds, the Delphi method gathers information and opinions, processes them, and then re-presents them to the same panel of people in order to refine them [5]. It allows for a group of people to collaboratively examine complex topics whilst simultaneously avoiding the disputes that would occur as a result of differences of opinion. With respect to animal welfare, the Delphi method has been used in a variety of ways to better understand the multifaceted problems associated with the subject matter (e.g. [6–9]).

This study sought to invite and report the opinions of selected equine professionals in Canada on the perceived welfare issues within the Canadian equine industry, their suspected prevalence, and the perceived root causes or drivers (henceforth referred to as “motives”) affecting these issues. Though the survey covered a number of topics regarding equine welfare, ultimately the objective was to determine if it was possible to reach a consensus of opinion regarding equine welfare issues.

## Method

This project was approved by the University of Guelph Research Ethics Board for compliance with federal guidelines for research involving human participants (REB #15DC024). All respondents were presented with a consent form prior to the survey and were required to accept before proceeding.

Three iterative rounds of a modified (online) Delphi survey, created and maintained through an online survey platform provided by Qualtrics (2016 Qualtrics LLC), were used to facilitate data collection (see S1 Appendix for the full survey). Questions used in the modified Delphi were written in consultation with a qualitative data expert (H.H.O.) to ensure non-biased open-ended wording that would encourage participant response. The three rounds of the survey were conducted online from February to April 2016, with each round lasting between two and three weeks, with deadlines extended to ensure at least half of previous round’s participants finished the round. All participants were provided with links to each round once the previous round was closed and the information was analyzed (see S1 Fig for schematic representation of the rounds). Only the question that prompted the participant to input their unique code number required a mandatory response; all other questions were optional.

### Equine professionals

Delphi methods typically utilize individuals who are considered informed about the topic (“experts” [4]). As such, this study targeted professionals (defined as people who had certification in their field of equine employment or who had been working in their equine-related job for more than ten years) in the following equine industry specializations: Equine researchers and welfare scientists, equine-focused/specialized veterinarians, equine dentists, farriers, equine nutritionists, certified riding coaches, racing jockeys and trainers, equine massage therapists, and those with an equine-related diploma, baccalaureate, or graduate degree. These groups were chosen based on their contribution to and involvement in the Canadian equine industry. Additionally, the Society for the Prevention of Cruelty to Animals (SPCA) offices in each Canadian province and territory were contacted to request the participation of agents who had experience dealing with cases of equine cruelty. While not all potential specializations were included, these groups were selected to allow for a wide breadth of experience from individuals who were actively involved in a variety of sectors of the horse industry.

Potential participants were gathered from those whose contact information was available publicly on their professional equine websites or in public advertisement directories (e.g. Equine Canada open directory of certified coaches) as well as personal contacts known professionally to the Primary Investigator (KM—11). They were contacted via email or telephone in order to gauge their interest in this project prior to the delivery of the consent form and first round of the survey. Potential participants were provided with a standardized information letter or phone script which outlined the nature of the study and what it entailed. A total of 215 individuals from across Canada were invited to participate in the project, of which 55 people agreed to participate (23% response rate). A total of eight contacted individuals declined to participate due to unavailability; all other contacted individuals did not respond to their invitation. Those who accepted the invitation were forwarded a link to the survey itself as well as an alphanumeric identification code that would serve as an identifier for subsequent rounds.

### Round 1

Round 1 questions were first tested on a pilot group (a class of 20, fourth-year undergraduate students in an equine management program) to ensure that the questions were well-understood, and to receive feedback on question composition (e.g. redundancy, word choice). After the pilot testing, the survey link was sent via email to the 55 participants who had agreed to participate. Participants were required to input their unique alphanumeric code to gain access to the survey and to ensure that subsequent rounds were only sent to those who had provided answers in the previous round.

The first round contained demographic questions, including participant age category, gender, province or territory of residence, involvement in the industry (type and duration), and equine-related education. Pre-determined options were provided for all categories except involvement in the industry, which was free text. This was then followed by questions that asked respondents to list welfare issues or concerns (at the individual horse level and at the industry level, similar to the distinction made in [6]) and how they might address these issues (again, at both the individual animal and industry levels). A welfare issue or concern was defined as “*anything <the participant> believes reflects or negatively affects a horse’s well-being*.*”* Individual-level welfare concerns were those that were considered by participants to be best addressed at the level of singular animals, while industry-level welfare concerns were those best addressed by the industry as a whole. All questions were open-ended such that survey respondents could list as many or as few points as they wished for each section. Finally, respondents were asked to comment on any aspect of their choosing regarding horse welfare in Canada and were given unlimited space to do so.

Data were exported from Qualtrics into Microsoft Excel and the responses were structurally coded. Categories of answers were created to combine similar responses (e.g. the category “*Horses being denied access to basic physical requirements*” included situations where horses were not being provided with food, water, shelter, or turnout), which were then presented to the survey respondents in the subsequent rounds. A total of 12 individual horse level welfare issues and 12 industry level welfare issues affecting horses across all sectors were determined from this round.

### Round 2

Round 2 focused on the perception of the issues categorized by qualitative analysis from Round 1. Based on the responses given in the first round, survey respondents were asked to rank the 12 individual-level welfare issues and 12 industry-level welfare issues based on their perceived importance with respect to the equine industry. They were then asked to indicate how prevalent they thought each of these issues was (on a scale of 0 to 5, with 0 being rare and 5 being prevalent in all industry sectors), and state via free text answer where they believed these issues were most often found within the industry (e.g. in a specific discipline such as the hunter/jumper sector, or a more broad geographical area such as western Canada). Survey respondents were also asked to list potential reasons or motives for the issues and concerns compiled in Round 1. As in the previous round, this question was open-ended, and survey respondents could provide as many or as few answers as they wished.

Data were exported from Qualtrics into Microsoft Excel for further analysis. Frequency tables were created for the questions which involved ranking of issues and Fleiss’ Kappa was calculated using Microsoft Excel (2016) to compare prevalence rankings. All open-ended questions were structurally coded using QSR International’s NVivo 10 qualitative data analysis Software (QSR International Pty Ltd. Version 10, 2012) and the number of coding references were tabulated. Categories of answers were created to combine similar responses (e.g. the motive category “*Lack of animal welfare legislation*” included both insufficient/lenient penalties as well as the existing legislation not being properly used).

### Round 3

Survey respondents were asked to rank the ways of addressing welfare concerns (14 at the individual horse level and 20 at the industry level) from most to least effective. Subsequently, they were asked to rank the motives compiled from the Round 2 from most to least important, with respect to their contribution to poor equine welfare.

### Consensus

There is no defined level of participant agreement considered to reflect “consensus” for Delphi studies [4]; levels of 51%, 70% and 80% have been suggested by [10–12] respectively. For the purposes of this paper “consensus” was considered to be reached at 70% or greater agreement, while values over 51% but less than 70% were considered “approaching consensus.”

## Results

### Equine professionals

Of the 55 survey respondents who agreed to participate, 34 (19 female and 15 male) completed Round 1, with at least one member from each of the industry groups targeted (distribution by category can be found in S2A Fig). The average participant was older than 45 years of age and had been involved in the industry for more than 30 years. The majority of participants (47%) lived in Ontario, with representatives from the following other provinces or territories in order of decreasing percentage: British Columbia, Quebec, Alberta, Nova Scotia, Saskatchewan, and Prince Edward Island (6/10 provinces, 0/3 territories).

The survey respondents were primarily involved in the industry through the English riding discipline (47% of survey respondents), while 29% of survey respondents were primarily involved in an "other" category (e.g. as a veterinarian). Survey respondents also indicated additional avenues in which they were involved in the industry, including horse or facility ownership (38%), working as an equine educator (15%) or selling equine-related products (9%). Survey respondents were also asked to list any certifications they possessed; the certification type and number of individuals who indicated they had certification can be found in Table 1.

**Table 1:** List of all certification types possessed by survey respondents (n = 34) and the number (%) of individuals with these certification types. A respondent could hold more than one certification.

Certification Type	Number of Survey respondents
**Veterinarian**	9 (26%)
**Equine-related diploma, baccalaureate, or graduate degree**	8 (23%)
**Accredited/Certified Farrier**	4 (12%)
**Coach Certification**	4 (12%)
**Certified Cruelty Investigator**	3 (9%)
**Registered Equine Massage Therapist**	3 (9%)
**Certified/Apprenticed Equine Dentist**	2 (6%)
**Veterinary Technician**	1 (3%)
**Horse Judging Certification**	1 (3%)
**Certified Veterinary Acupuncturist**	1 (3%)

### Round 1

Participants’ responses from Round 1 were categorized into 12 welfare issues believed to be experienced by horses in the Canadian industry at the individual horse level and an additional 12 (with minimal duplication) that were believed to be experienced by horses at the industry level (Table 2). Ignorance or lack of knowledge, overpopulation, and lack of long-term planning were three issues that appeared in both sections.

**Table 2:** Categories of Canadian equine industry welfare issues at the individual horse and industry level as indicated by a panel of equine professionals (n = 34) in Round 1 of a modified Delphi survey. Issues with duplication between individual and industry level indicated in italics with remaining issues listed in no particular order.

Welfare Issues at the Individual horse Level	Welfare Issues at the Industry Level
*Too many horses/unregulated breeding*	*Overpopulation of horses (including lack of breeding control*, *unwanted animals)*
*Lack of knowledge or education (including incorrect information being perpetuated*, *little value put on evidence-based information*, *novice owners/owners who don’t appreciate time and financial commitment required by horses)*	*Ignorance and lack of knowledge (especially related to horse learning theory and horse behaviour)*
*Lack of long-term planning or end of life care planning by owners*	*Lack of long-term planning or end of life care planning by owners*
Inappropriate training practices (including excessive use of aids, spike poles for jumpers, overworking horses, working horses at a level beyond their physical abilities, having unreasonable expectations, training horses too young, soring of gaited horses)	Perpetuation of outdated or disadvantageous practices
Inappropriate drug use (including; Lasix in horse racing, joint injections, tail blocking/nerving, misuse of medication, "masking" lameness through painkillers)	Horse slaughter and horses housed at feedlots
Breeding for aesthetic but detrimental traits	Lack of accountability by individuals(e.g. professionals, owners, including veterinarians not reporting)
Horses being denied access to basic physical requirements (e.g. food, water, shelter, turnout)	Lack of standards of care for horses
Horses being denied access to important psychological resources (e.g. companionship/social interaction)	Lack of regulation at the industry level for practices detrimental to welfare (e.g. rules for drug use in competitions)
Improper dietary practices (including overfeeding and obesity, incorrect feeding practices)	Lack of regulation at the government level supporting equine welfare
Lack of proper professional care (i.e. owner not sourcing professional veterinarians, dentists, farriers, etc. to meet their horses’ needs)	Lack of knowledge transfer from research to the horse owning community
Lack of daily or attentive monitoring (including taking preventative measures)	Poor biosecurity practices
Lack of skilled personnel within the industry (including lack of necessary training programs)	Poor public image of the equine industry

Additionally, survey respondents identified 14 potential methods of addressing equine welfare concerns at the individual horse level, and an additional 20 methods of addressing equine welfare concerns at the industry level (Table 3).

**Table 3:** Methods of addressing equine welfare concerns at the individual horse and industry level as indicated by a panel of equine professionals (n = 34) in Round 1 of a modified Delphi survey. Methods with overlap between individual and industry level indicated in italics with remaining methods listed in no particular order.

Individual	Industry
*Education (e*.*g*. *more centralized*, *increased awareness*, *more reliable sources*, *pre-purchase knowledge)*	*Education for all people dealing with horses (e*.*g*. *owners*, *farriers*, *feed companies)—includes continuing education priorities for organization members*, *targeted public education*, *responsible horse ownership*, *teaching through welfare advocates*, *education on horse needs*
*Change rules and regulations issued by equine associations (e*.*g*. *redefine competition judging standards to better reflect natural horse behaviours)*	*Alter the way horses are judged (e*.*g*. *focusing more on conformation and sound movement rather than imposed aesthetics)*
B*etter understanding of equine behaviour and behavioural cues*	*Better understanding of equine behaviour and learning theory*
Utilize veterinary equipment to determine if procedures are necessary (e.g. make use of ultrasound equipment before joint injections are performed)	Collect industry data to serve in the creation of benchmarks for acceptable standards (e.g. career duration of competitive horses)
Contact/report cases to a regulation body (e.g. SPCA, Ontario Racing Commission/Association)	Increased control of drug usage in the competition sector (e.g. harsher penalties, mandatory intermittent drug testing, increased accountability)
Make proper horse care the primary goal (as opposed to winning, for example)	Cooperation within the industry to work towards common goals (e.g. creating a united front when approaching the government for assistance or support)
Industry stake holder involvement (e.g. initiate educational sessions for horse owners)	Require horse owners to be licensed/registered before owning animals (i.e. ensure proper knowledge regarding horse care)
Regulate breeding	Records of sales and transfers of ownership of horses
Strengthen and enforce animal welfare legislation	Restriction or banning of live horses exported from Canada for the purposes of slaughter
Allow horses more access to physical requirements (e.g. food, water, shelter, turnout)	Increased number of officials at competition events (e.g. required veterinary checks before, during, and after competitions for all animals)
Change perception of practices (e.g. what constitutes a good trainer)	Regular inspections of facilities (i.e. special attention paid to training methods)
Daily checks of animals and their housing systems (e.g. fence checks)	Decreased incentives for utilizing young horses in competition (e.g. age restrictions)
Better communication between equine professionals (veterinarians, farriers, nutritionists, dentists, etc.) and owners	Development of evidence-based tools in order to better assess equine welfare
Consistent routine care (e.g. hoof trimming, dental exams)	Restriction or banning of live horses imported to Canada for the purposes of slaughter
	Mandatory welfare training for officials who oversee horse welfare (e.g. educating policy makers—especially in the government—regarding horse needs)
	Equine associations acting as leaders and advocates of good practice
	Increased control of horse slaughter (e.g. better awareness, harsher penalties for fraudulent dealings, stricter drug testing, industry-recognized identification system)
	The creation of a universal definition of equine welfare
	Increased provincial and/or federal regulation
	Changes in legislation for competition horses (e.g. increased safety of horses competing)

#### Open comments regarding equine welfare in Canadian industry

In the final section of Round 1 only, survey respondents were invited to comment on any aspect of welfare as it related to the Canadian equine industry. Of the 20 comments, husbandry, education and legislation concerns were the three themes with the highest number of references (7, 5, and 4 respectively). Comments related to equitation, communication, use of professionals, hoarding of horses, horse lifestyle and different equine sectors were also present. There were five instances where participants stated that they felt the welfare of horses in Canada was "generally good", and two instances where they felt it was "generally poor."

### Round 2

Of the 34 respondents who completed the first round, 24 completed Round 2 (distribution by category can be found in S2B Fig), though some survey respondents chose not to answer some of the questions. Using the issues generated in Round 1, survey respondents were asked to rank the issues from most (1) to least (12) important for both the individual- and industry-level problems. Due to a lack of agreement among survey respondents for individual ranks, the frequency with which each issue was ranked in the participant’s top six was calculated instead. This was calculated for both individual-level issues (Table 4) and industry-level issues (Table 5).

**Table 4:** Frequency of individual-level welfare issues as ranked by equine professionals (n = 24) in Round 2 of a modified Delphi survey.

Issues indicated in Round 1^a^ at the Individual horse level	Frequency Issue Ranked in top 6 % (number of respondents)^b^
Horses being denied access to important psychological resources	71.43% (15)
Inappropriate drug use	71.43% (15)
Horses being denied access to basic physical requirements	66.67% (14)
Lack of proper professional care	66.67% (14)
Inappropriate training practices	61.90% (13)
Lack of knowledge or education	61.90% (13)
Overpopulation	52.38% (11)
Improper dietary practices	52.38% (11)
Lack of long-term planning or end of life care planning	28.57% (6)
Breeding for aesthetics	23.81% (5)
Lack of skilled personnel within the industry	23.81% (5)
Lack of daily or attentive monitoring	19.05% (4)

^a^See Table 2 for full description of issues

^b^ Due to a lack of agreement, the frequency which the issues were ranked by survey respondents (n = 21) in the top six is presented. Three respondents chose not to answer this section. Lightly shaded areas indicate values that approached consensus (>51%). Darker shaded areas indicate consensus (>70%).

**Table 5:** Frequency of industry level welfare issues as ranked by equine professionals (n = 24) in Round 2 of a modified Delphi survey.

Issues indicated in Round 1^a^ at the Industry Level	Frequency Issue Ranked 1–6 % (number of respondents)^b^
Ignorance/ lack of knowledge	90.91% (20)
Overpopulation of horses	81.82% (18)
Lack of regulation at the industry level	68.18% (15)
Horse slaughter and horses at feedlots	63.64% (14)
Lack of accountability	54.55% (12)
Perpetuation of outdated or disadvantageous practices	54.55% (12)
Lack of standards of care for horses	45.45% (10)
Lack of regulation at the government level	36.36% (8)
Lack of knowledge transfer from research	36.36% (8)
Lack of long-term planning	31.82% (7)
Poor biosecurity practices	22.73% (5)
Poor public image of the equine industry	13.64% (3)

^a^See Table 2 for full description of issues

^b^ Due to a lack of agreement, the frequency which the issues were ranked by survey respondents (n = 22) in the top six is presented. Two respondents chose not to answer this section. Lightly shaded areas indicate values that approached consensus (>51%). Darker shaded areas indicate consensus (>70%).

The perceived prevalence of each issue was ranked with zero representing “rare” and five representing “prevalent in all industry sectors.” At the individual horse level, over 40% of survey respondents indicated that they felt inappropriate drug use merited a prevalence rank of 4 out of 5 and assigned both horses being denied access to physical requirements and lack of daily or attentive monitoring a prevalence rank of 2. At the industry level, over 40% of survey respondents assigned the perpetuation of outdated or disadvantageous practices a prevalence rank of 5/5, ignorance/lack of knowledge a rank of 4/5 and poor public image of the equine industry a rank of 2/5. Results from the Fleiss’ Kappa (κ = 0.01) showed only slight agreement between participants.

When asked where a particular welfare issue or concern was most evident within the industry, survey respondents’ answers were grouped into three major categories: horse-related “specialities” (e.g. a particular discipline), geographical locations (e.g. Eastern Canada—Ontario and Quebec) and a “don’t know” category. With respect to discipline, participants most frequently reported that issues were found within the racing industry [35 references] and English disciplines [18 references]. The majority of answers, however, were that welfare problems existed in all sectors [72 references]. There were a total of 44 references to a specific part of Canada (e.g. Eastern Canada [22 references]) as well as 21 references to "remote areas” regardless of province or territory. While the majority of references indicated a high prevalence in Eastern Canada, references to Western Canada [9 references], the Maritime provinces (Nova Scotia, New Brunswick, Newfoundland and Labrador, and Prince Edward Island) [5 references], and Ontario [4 references] were also made. There were 12 instances where survey respondents indicated that they did not know or were unsure of the prevalence of a particular issue. Each of these instances occurred for a unique issue (two at the individual horse level and ten at the industry level). Finally, survey respondents identified 14 potential motives for the welfare issues listed from Round 1 (Table 6).

**Table 6:** Potential motives for equine welfare issues as suggested by equine professionals (n = 34) in Round 2 of a modified Delphi survey. Motives are listed in no particular order.

Potential Motives Suggested by Expert Panel
Lack of animal welfare legislation (insufficient and lenient penalties, not properly used)
Human convenience (e.g. providing concentrate feed in discrete meals)
Limited equine research
Lack of resources involved in investigation and prosecution (e.g. in equine abuse/neglect cases)
Financial gain (e.g. associated with competition, desire to win)
Anthropomorphism (attributing human emotions to animals)
Ignorance/lack of education (at the government level)
Tradition
Willful neglect and abuse
Ignorance/lack of education (at the owner level)—includes the lack of knowledge that horses are a lifetime commitment
Financial difficulties (lack of resources)
Lack of leadership in the equine community
Perception of horses as disposable commodities
Lack of access to professionals (e.g. in remote areas)

### Round 3

Of the 24 people who completed the second round, 14 completed the third round (distribution by category can be found in S2C Fig). The majority of these participants were female (43%), over 45 years of age (64%), resided in Ontario (50%), and had been involved in the industry for more than 30 years (57%). These individuals most actively participated in the English riding discipline, but members of the horse racing and breed competitions were also present.

In the third round, survey respondents were asked to rank the effectiveness of strategies of addressing welfare concerns at the individual horse and industry levels. Due to a lack of survey respondent agreement, the frequency with which each method was ranked in the participant’s top seven (in the case of individual horse level; Table 7) and top ten (in the case of industry level; Table 8) were calculated instead.

**Table 7:** Methods of addressing equine welfare concerns at the individual horse level identified in Round 1 and the frequency which they were ranked in the top seven by equine professionals (n = 14) in Round 3 of a modified Delphi survey.

Methods of addressing equine welfare concerns (individual horse level)	Frequency Method Ranked in top 7 % (number of respondents)^a^
Allow horses to have more access to physical requirements	92.86% (13)
Make proper horse care the primary goal	85.71% (12)
Consistent routine care	78.57% (11)
Strengthen and enforce animal welfare legislation	57.14% (8)
Education	57.14% (8)
Daily checks of animals and their housing systems	50.00% (7)
Change perception of practices	50.00% (7)
Better communication between equine professionals	50.00% (7)
Better understanding of equine behaviour and behavioural cues	42.86% (6)
Industry stake holder involvement	35.71% (5)
Regulate breeding	28.57% (4)
Utilize veterinary equipment to determine if procedures are necessary	28.57% (4)
Contact/report cases to a regulation body	28.57% (4)
Change rules and regulations issued by equine associations	14.29% (2)

^a^Lightly shaded areas indicate values that approach consensus (>51%). Darker shaded areas indicate consensus (>70%).

**Table 8:** Methods of addressing equine welfare concerns at the industry level identified in Round 1 and the frequency which they were ranked in the top ten by equine professionals (n = 14) in Round 3 of a modified Delphi survey.

Methods of addressing equine welfare concerns (industry level)	Frequency Method Ranked in top 10 % (number of respondents)^a^
Education for all people dealing with horses (e.g. owners, farriers, feed companies)	92.86% (13)
Better understanding of equine behaviour and learning theory	85.71% (12)
Mandatory welfare training for officials who oversee horse welfare	78.57% (11)
Collect industry data to serve in the creation of benchmarks for acceptable standards	71.43% (10)
Increased control of drug usage in the competition sector	71.43% (10)
Decreased incentives for utilizing young horses in competition	64.29% (9)
Development of evidence-based tools in order to better assess equine welfare	64.29% (9)
Cooperation within the industry to work towards common goals	64.29% (9)
Increased control of horse slaughter	50.00% (7)
Require horse owners to be licensed/registered before owning animals	50.00% (7)
Alter the way horses are judged	50.00% (7)
Records of sales and transfers of ownership of horses	42.86% (6)
Restriction or banning of live horses exported from Canada for the purposes of slaughter	35.71% (5)
The creation of a universal definition of equine welfare	35.71% (5)
Regular inspections of facilities	28.57% (4)
Restriction or banning of live horses imported to Canada for the purposes of slaughter	28.57% (4)
Equine associations acting as leaders and advocates of good practice	21.43% (3)
Increased number of officials at competition events	21.43% (3)
Increased provincial and/or federal regulation	21.43% (3)
Changes in legislation for competition horses	21.43% (3)

^a^Lightly shaded areas indicate values that approached consensus (>51%). Darker shaded areas indicate consensus (>70%).

Additionally, survey respondents were asked to rank the motives of welfare issues/concerns. Again, due to a lack of survey respondent agreement, the frequency with which each motive was ranked in the respondent’s top seven was calculated instead (Table 9).

**Table 9:** Potential motives for equine welfare issues identified in Round 2 and the frequency which they were ranked in the top seven by equine professionals (n = 14) in Round 3 of a modified Delphi survey.

Potential Motives Suggested by Expert Panel	Frequency Motive Ranked in top 7 % (number of respondents)^a^
Ignorance/lack of education (at the owner level)	85.71% (12)
Human convenience	78.57% (11)
Financial gain	64.29% (9)
Financial difficulties (lack of resources)	64.29% (9)
Lack of animal welfare legislation	57.14% (8)
Willful neglect and abuse	50.00% (7)
Lack of resources involved in investigation and prosecution	50.00% (7)
Anthropomorphism	50.00% (7)
Ignorance/lack of education (at the government level)	50.00% (7)
Tradition	42.86% (6)
Limited equine research	42.86% (6)
Perception of horses as disposable commodities	42.86% (6)
Lack of leadership in the equine community	14.29% (2)
Lack of access to professionals (e.g. in remote areas)	7.14% (1)

^a^Lightly shaded areas indicate values that approached consensus (>51%). Darker shaded areas indicate consensus (>70%).

## Discussion

### Delphi survey response and demographics

The use of multiple rounds of the Delphi technique allowed for survey respondents to provide insight from their own experiences and perceptions of the Canadian equine industry during the “brainstorming” portions and refine these answers with input from other respondents in subsequent rounds. While not all user groups were included, the professionals chosen to participate in the survey represented the most predominant user groups in the industry. For example, while there was no representative of the horse meat industry, this sector represents only 0.7% of horses, while horses used for riding and driving comprise 36% of the Canadian horse industry [2]. The initial survey respondents had a near even split by gender, but exhibited a bias towards residents of Eastern Canada and professionals involved in English riding disciplines. This is comparable to the demographic spread of the Canadian equine industry [2].

Despite the decrease in participation between rounds, the representation among the categories remained strikingly similar (S2 Fig), survey respondents demonstrated a willingness to provide and share their experiences in the Canadian equine industry, which resulted in a number of unique welfare issues at both the individual horse and industry level (with minimal duplication), and the number of ideas to address welfare issues (again, with differences at individual horse and industry levels). This willingness to critically evaluate their own industry is promising for future studies in this area.

Experiences volunteered in the open comments section, however, were sparse, which may have been a result of the number of questions in the modified Delphi or the multiple opportunities within the survey for the respondents to share their thoughts. Despite being asked to describe the potential problems within the industry, several participants suggested that the overall welfare of horses in the equine industry was “good”, which is supported in later rounds by the prevailing sense that while the industry as a whole has much to improve on, there are individuals within it who care about the welfare of their animals. A similar attitude towards equine welfare was also found by McNeill *et al*. [13] for surveyed South Dakota equine industry participants.

### Welfare issues at the individual horse and industry level

With respect to the welfare issues at the individual horse level, inappropriate drug use and horses being denied access to psychological resources (e.g. companionship) received consensus as issues of importance. While participants believed that all horses were at risk, an estimated 23.6% of horses in the Canadian equine industry are used in some form of competitive sport (e.g. racing [2]), which—alongside the high-profile nature of competition horses—may account for the attention given to inappropriate drug use. The effect of the lack of psychological resources on horse welfare is more difficult to measure than the lack of physical resources such as food, water and shelter. Despite the belief that horses do have behavioural needs which should be attended to when managing their living environment, the perception remains that these needs are not regularly provided for [13, 14]. Conversely, breeding for aesthetics, lack of daily or attentive monitoring, lack of skilled personnel within the industry, and a lack of long term planning were considered the issues of lowest importance. Lack of long term planning could pertain to owners who do not intend to keep young horses as long as they do, and those who do not expect to have to care for their horses into old age (and perhaps have not set aside funds to deal with failing health in geriatric animals).

By comparison, at the industry level, ignorance/lack of knowledge and overpopulation were considered of greatest importance. Ignorance or lack of knowledge is a pervasive theme in many studies [7, 15, 16, 17], and is discussed more fully below. It is unclear what the specific cause or causes of overpopulation is thought to be (if it is not lack of long term planning), and given that 22.8% of horses in the Canadian equine industry are young horses not yet in work [2], this warrants further investigation in future studies. At the industry level, poor biosecurity practices and poor public image were considered of least importance. With respect to biosecurity practices, Schemann *et al*. [18] noted that several factors (including farm size and whether or not an individual farm had horses contract an infectious disease) affected a participant’s perception of the effectiveness of certain biosecurity measures. It is possible that of those surveyed in this study, past experiences with infectious diseases positively affected their perceptions of current biosecurity practices, prompting them to rank it of lower importance. Poor public image has mostly affected participation in sporting events. Images of catastrophic breakdowns of race horses, for example, may cause race attendance to decrease, which in turn would decrease betting (and thus revenue for the racetracks which support the industry). While this is nowhere near the level of damage that poor public perception can cause the food animal industries [19], it can still affect the livelihoods of those involved in equine industry sectors which provide entertainment. Though poor public image was not a concern in this study, findings by Derisoud *et al*. [14] noted a significant difference between actual and perceived management practices when comparing Canadian horse owners and non-horse owning industry participants. Despite this difference, however, the impact these perceptions have on horse welfare is arguably low, which is likely why it was consistently of least importance.

### Perceived prevalence and location of welfare issues

The widespread perception of inappropriate drug use is a noteworthy result since—as previously stated—the competition sector of the Canadian equine industry comprises fewer than 25% of all animals [2]. It is unclear if, by assigning this high prevalence, participants believed that the majority of competitive horses are given drugs, or if this high prevalence also included drug use in non-competitive horses. The comments collected during Round 1 suggest that the focus of this category was competitive horses; however, comments such as “misuse of medication” and “masking lameness through painkillers” could apply to horses in all sectors. A study could not be found examining different industry participants’ perceptions—either professionals or owners—of inappropriate drug use versus drug abuse with respect to horses and this would merit further investigation.

There appeared to be minimal duplication between issues deemed important and those deemed the most prevalent. This may be because some issues perceived to be more commonly seen within the industry (e.g. perpetuation of outdated or disadvantageous practices) lack the perceived severity or long-lasting consequences of those ranked more important (e.g. drug abuse [20, 21]).

The belief that horses in all sectors are at risk for welfare issues (not just horses of a particular discipline) may be a by-product of the belief that ignorance is an industry-wide problem (and because of this ignorance, all horses are at risk). Alternatively, the phrasing of the question (horse-related “speciality” and a geographical location) may have may have influenced the answers. These answers differ from those given by participants in the Irish equine industry, who highlighted unregulated fairs, unlicensed races, and disposal locations as areas where welfare was most likely to be compromised [7]. It is worth noting, however, that in both cases, areas in which regulation or oversight is lacking are singled out, suggesting that welfare issues may be more likely to occur where the likelihood of ramifications is also low. Though participants in the British equine industry also indicated race horses were at a higher risk for welfare concerns [15], the only other group mentioned were traveller’s horses, which the Canadian equine industry does not have.

Participants in this survey were strongly biased towards the English riding disciplines and the equine industry in Eastern Canadian provinces. Differences in the distribution of horses by use are visible in the 2010 Canadian Horse Industry Profile Survey [2], but the extent to which these differences result in vastly different welfare issues is yet unknown. Going forward, province-specific surveys may be necessary to determine if a country-wide approach to improving equine welfare will be viable, or if a provincial- or individual sector-level approach is best-suited to tackling the industry-wide issues.

### Perceived ignorance in the Canadian equine industry

Ignorance has been defined as of a state of not knowing (lack of knowledge [22]; and as a lack of self-awareness which results in a refusal to learn more (“closed ignorance” [23]). Delphi survey participants referenced both of these definitions, which suggests not only a problem of available knowledge but also a reluctance to learn. The idea that there are knowledgeable individuals within the industry but that the industry as a whole suffers from ignorance and lack of knowledge was referenced in all three rounds. Ignorance and lack of education were suggested as issues that place individual horses and the industry at risk through the perpetuation of outdated practices, depriving horses of physical resources, etc. (see Table 2). Ignorance and lack of education were also suggested as motives for decreased welfare of horses individually and industry-wide for reasons such as money (not being able to afford education), pride, etc. (see Table 2). It is possible that, despite having knowledgeable professionals or individuals within the industry (e.g. owners), their knowledge is not being circulated or passed on in a way that would combat industry-wide ignorance. In a similar study by Horseman *et al*. [16] survey respondents indicated that owners were not seeking advice at all or from the appropriately qualified people and highlighted this as an important cause of welfare issues in Great Britain.

Themes of educating and self-governance (or owner responsibility) emerged at both the individual horse and industry level, as the data suggest that Canadian equine professionals value educating people so they can help themselves more than an increase in equine association or government involvement. At both levels, all methods of addressing welfare concerns which would involve an increase in regulation or influence from a governing body (even an equine one) were not considered to be effective, which suggests that equine professionals do not believe introducing more or new rules will positively impact the welfare of horses individually or as a whole. The only instance where more control was suggested was with respect to drug administration, which is unsurprising given that participants ranked inappropriate drug use as an important issue in Round 2. Instead, participants focused on all the options that would provide greater education for individual horse owners as well as any people who are responsible for the care of horses. In Round 2, participants indicated that they felt that while ignorance was an industry-wide problem, there was not a shortage of knowledgeable individuals. These results suggest that participants believed it would be better for horses in the Canadian equine industry if more emphasis was placed on educating caretakers and gathering information (e.g. on equine behaviour) in an effort to provide better care in the future. This is supported by perceived prevalence of ignorance and the perpetuation of outdated or disadvantageous practices, and by motives which achieved consensus as most important (ignorance/lack of education [at the owner level] and human convenience) and least important (lack of leadership in the equine community and lack of access to professionals). Ignorance, among others, is also cited as a potential cause of poor equine welfare in the Irish equine industry [7], the British equine industry [15], and the Australian recreational horse industry [17] suggesting a more widespread belief by industry participants that those who perpetuate poor equine welfare simply do not know any better.

### Comparison of Canadian equine industry to other countries

Alongside ignorance, a study by McNeill *et al*. [13] identified the high cost of horse care, “poor horsemanship” and dental problems as major equine welfare issues in South Dakota. While the cost of horse care was not considered to be a welfare issue by Canadian Delphi participants, the role that finances play in equine welfare was alluded to when discussing motives. Canadian participants also highlighted the importance of long-term planning with respect to horse care, with financial elements being one aspect. The differences in the importance of horse care costs may be reflective of how much of the participants’ income came from their horses. According to the 2010 Canadian Industry Profile [2], horses were the major source of household income for only 8% percent of industry participants, indicating that involvement with horses is mainly a leisure activity, with the financial resources for horse care coming mainly from outside sources. As a result, the financial burden may not be a serious concern for many members of the Canadian equine industry.

Participants in Horseman’s [15] survey of British industry participants divided their welfare issues into three categories (health-, management-, and riding-related), and raised many of the same issues as Canadian participants, such as the denial of physical resources (particularly feed) and inappropriate training methods. There were, however, issues that are unique to each country. Tethering and “fly grazing” (grazing animals on land without permission) are two practices that were not suggested as issues by Canadian participants. In contrast, British participants did not question the use of professionals, nor raise issues related to horses at slaughter plants or feedlots. This is likely due to the fact that there is no recognized horse meat market in the UK, while Canada struggles with accommodating the horse slaughter industry from the USA. Further differences also appeared when the results from this study were compared to the welfare problems of the Irish equine industry. Equine industry participants in Ireland cited overpopulation, identification, import and export, owner irresponsibility and the absence of humane and “attractive” methods of euthanasia as issues of importance with respect to welfare [24]. The focus on overpopulation of equids and resulting sub-issues (such as the import and export of horses) suggests an entirely different industry profile in Ireland. The majority of horses in Ireland are categorized as racing or sport horses, and as such there is a greater focus on horse identification and ownership due to the high potential for horses to change owners [24]. In contrast, the Canadian equine industry is composed of competition horses (of which race horses make up only 5.4%), young horses, breeding stock and horses used solely for pleasure or driving [2]. Both the diversity of horse “specialities” as well as the differences in available land space in each country have contributed to the growth of two industries which use horses in different ways. It can be suggested that the differences in focus indicate that the equine industry is not universal and requires understanding, management, and monitoring unique to its location or country.

### Addressing welfare issues in the industry

It remains unclear which issues most severely compromise welfare and what proportion of the equine population these issues affect. Survey participants were largely in favour of educational solutions to welfare issues and less supportive of measures which involved increased regulations and government involvement. While this is in line with their view of ignorance as the most contributing factor to equine welfare issues, participants also noted the importance of human convenience and financial elements involved in horse care. Even if ignorance or lack of knowledge can be “eliminated” with the assistance of increased educational programs, it may not be enough to see human behavioural change if other motives are not addressed. Surprisingly, participants had no suggestions (aside from punitive ones), that could change the behaviour of people who contributed to poor equine welfare for reasons beyond their own ignorance. The study of changing human behaviour recognizes three key components: capacity, motivation, and opportunity [25]. While improving education can increase a person’s capacity to change, they must also want to change (motivation) and have the ability to make the desires change (opportunity). In order to affect meaningful change, all components must be addressed, which is something worth further investigation in future surveys.

While participants were strongly interested in better education for all owners, a “one size fits all” approach may not work in an industry as diverse as that of the Canadian equine industry. Determining not only what education owners need, but also how best to ensure the educational material reaches them will be a challenging task in such a fragmented industry. The degree to which other potential methods of improving welfare may reach different industry sectors is also questionable. For some groups, such as competition or sport horses, industry body oversight is a regular occurrence, and can be used to enforce changes in behaviour. For other groups, such as the “backyard horse owners”, it may be difficult to determine how involved they are with changing industry practices. Additionally, the wide range of issues and minimal duplication suggests that welfare can be improved on many different levels (from the individual horse to the industry level). Participants were not asked to compare the methods of addressing welfare at these two levels, but evaluating which should be addressed first (the individual horse or the industry) warrants further investigation.

### Limitations to this study

Throughout the survey rounds, a representative from each of the targeted expert groups participated, with the exception of equine nutritionists who did not have a representative in Rounds 2 and 3. It is possible that, had there been an equine nutritionist present in Rounds 2 and 3, the individual-level issue regarding improper dietary practices may have achieved a higher rank. The effect of their absence is likely to be limited, however, as there were no specific questions regarding the components of balanced nutrition within the survey. Moreover, Delphi surveys treat individuals as a singular group (“experts”), which is not intended to be completely representative. Despite this, awareness of the potential biases of this group of equine professionals is still important to consider. Respondents were most commonly based in Eastern Canadian provinces and involved in English riding disciplines, and as such, their opinions likely mean that the information gathered is most applicable to the sectors of the industry they are most familiar with. While this study retained an acceptable response rate according to Sumison’s [11] criteria (70%), respondent rate dropped to 58% during Round 3, perhaps due to the timing or a decrease in participant interest. In contrast, Collins *et al*. [7] was able to maintain full participation of 44 respondents over the course of a three-round Delphi, despite the relatively comparable time span (November to February). Researchers noted, however, that the time period was chosen because it fell during the least busy season in the Irish equine industry, which may have impacted the response rate. The diversity of opinions was maintained throughout the rounds, despite some professional groups only having one representative member and some groups having no representation at all (e.g. horse meat buyers). Even so, the small sample size, particularly in the third round, indicates that results from this study should be taken cautiously. The repeated return to themes of ignorance warrant further investigation in a study with a larger sample size to determine if they emerge again. Many identified issues did not reach consensus within each category, which may have been due to the large number of issues presented (and thus number of options presented). While survey respondents were allowed to move the text box containing each issue around within the interface, determining the relative importance of twelve issues (and fourteen motives) with respect to each other was likely still a challenge. In future studies, questions asking survey respondents to select their “top three” issues of importance may achieve greater consensus, as well as indicate a true ranking (as opposed to a selection of issues that most often were ranked in the top five). The results from the current study were unable to determine which issues were the “most” important; only those which were relatively more important than others.

The exploratory and general nature of this study meant that data was often categorized to both limit the number of potential responses in an effort to conserve survey space and to approach the topic as broadly as possible. In doing so, however, participants may not have been able to appropriately rank these grouped issues if they were divided on the different elements of the issue. For example, “deprivation of physical resources” was one of the categories of welfare issues, which contained not only lack of food or water, but also turnout. When discussing the importance of this issue or its prevalence, participants may have felt that horses were provided adequate food and water, but that many horses were not provided sufficient turnout. As a result, participants may have awarded a “middle” rank for this issue to account for the low prevalence of one portion (food and water) but the high perceived prevalence of another (turnout). In order to properly evaluate specific welfare issues (e.g. lack of appropriate turnout) in the future, issues would need to be left individualized, as seen in Horseman [15]. In this way, better distinctions could be made, and a better consensus may be able to be reached.

One of the biggest limitations of this study is the paucity of up-to-date data about the Canadian equine industry to compare perceptions to. The most recent industry report was conducted in 2010 [2] and was entirely self-reported by individuals who were willing to complete the survey and were exposed to it through involvement in the sport horse industry or Equestrian Canada. More industry data, beyond the number of animals appearing in census documents, is required in order to truly understand any differences between the perceptions of the equine industry and its realities. A centralization of existing data collected on horses (e.g. drug test results from competition horses, veterinarian data, reports from officers of the Society for the Prevention of Cruelty to Animals) is the first step to helping bolster the knowledge base. To further understand the prevalence of other welfare issues, other standardized testing methods (e.g. on-farm welfare assessments) including owner surveys would be best employed.

## Conclusions

Though consensus was difficult to reach with such a broad and diverse group of professionals, clear themes emerged from this exploratory Delphi study. The concepts of “ignorance” and “lack of knowledge” persisted throughout all three rounds, cited as both potential welfare issues and motives for poor welfare situations. Respondents indicated solutions to welfare problems at both the individual horse and industry level which relied on external influence (e.g. government bodies) as the least effective, instead supporting solutions which focused on acquiring a better understanding of equine behaviour and sharing that knowledge with all those who interact with horses. This study suggests that in order to improve the welfare of equines in the Canadian industry, a greater effort is required to help educate its participants such that, through better daily routine care and management, higher standards of welfare can be attained. A strong emphasis on knowledge, rather than regulations or policing, is a clear indicator that equine professionals feel that ignorance is the biggest problem within the Canadian equine industry with respect to welfare.

While public pressure has influenced welfare-friendly movements in other livestock species, the lack of this pressure combined with the equine industry’s diversity and fragmented nature make it challenging to deal with the industry as a whole. Though consensus was difficult to achieve in this study, the answers given by equine professionals—through open comments as well as ranking questions—help to provide baseline data for a better understanding of the attitudes and perceptions held by professionals in the Canadian equine industry with respect to welfare. Establishing baseline attitudes is valuable for determining changes in perception over time, particularly in response to efforts to improve welfare. Information regarding what long-time industry participants and professionals consider “welfare concerns”, how they feel they should be addressed, as well as what motivates people to expose horses to these situations is vital in determining the best strategies to implement improvements to equine welfare both individually and industry-wide in Canada. The use of the Delphi method assisted in the determination of perceived “important issues”, which can direct future research not only to determine the effects these welfare-threatening situations have on horses but also potential strategies to improve or remove these situations all together.

## Supporting information

S1 Appendix Delphi survey questions rounds 1–3 as presented to participants. (PDF)

S2 Appendix Quantitative data for rankings. (XLSX)

S1 Fig Schematic representation of the subject matter and flow of each modified Delphi round. (PDF)

S2 Fig Distribution of respondents by professional category in Round 1 (A: n = 34), Round 2 (B: n = 24) and Round 3 (C: n = 14) of a Delphi survey. (XLSX)

## References

[1] National Farm Animal Care Council. Code of Practice for the Care and Handling of Equines. 2013. http://www.nfacc.ca/pdfs/codes/equine_code_of_practice.pdf

[2] Evans V. Canadian Horse Industry Profile Survey. Equine Canada. 2010. https://www.equestrian.ca/industry/about

[3] Lagerkvist CL and Hess S. A meta-analysis of consumer willingness to pay for farm animal welfare. Eur Rev Agric Econ 2011;38: 55–78.

[4] Hasson F, Keeney S and McKenna H. Research guidelines for the Delphi survey technique. 1 2000;32: 1008–1015. 11095242

[5] Okoli C and Pawlowski SD. The Delphi method as a research tool: an example, design considerations and applications. Information & Management 2004;42: 15–29.

[6] Whay HR, Main DCJ, Green LE and Webster AJF. Animal-based measures for the assessment of welfare state of dairy cattle, pigs and laying hens: consensus of expert opinion. Animal Welfare 2003; 12: 205–217.

[7] Collins J, Hanlon A, More SJ, Wall PG and Duggan V. Policy Delphi with vignette methodology as a tool to evaluate the perception of equine welfare. Vet J 2009;18: 63–69. 10.1016/j.tvjl.2009.03.012 19375962

[8] Collins J, Hanlon A, More SJ, Wall PG, Kennedy J and Duggan V. Evaluation of current equine welfare issues in Ireland: Causes, desirability, feasibility and means of raising standards. Equine Vet J 2010; 42: 105–113. 10.2746/042516409X471458 20156244

[9] Collins J, More SJ, Hanlon A, Wall PG, McKenzie K and Duggan V. Use of qualitative methods to identify solutions to selected equine welfare problems in Ireland. Vet Rec 2012;170: 442–446. 10.1136/vr.100281 22331502

[10] McKenna HP. The Delphi technique: a worthwhile approach for nursing? 1 1994; 19: 1221–1225. 10.1111/j.1365-2648.1994.tb01207.x 7930104

[11] Sumision T. The Delphi technique: an adaptive research tool. Br J Occup Ther 1998; 61: 153–156.

[12] Green B, Jones M, Hughes D and Williams A. Applying the Delphi technique in a study of GP’s information requirements. Health Soc Care Community 1999;7: 198–205. 11560634 10.1046/j.1365-2524.1999.00176.x

[13] McNeill LR, Bott RC, Mastellar SL, Djira G, Carroll HK. Perceptions of Equid Well Being Well-Being in South Dakota. J Appl Anim Welf Sci 2018;21:1 40–68, 10.1080/10888705.2017.1372199 28910169

[14] Derisoud E, Nakonechny L and Merkies K. An industry view of perception and practice of equine management in Canada. J Vet Behav 2016;15 10.1016/j.jveb.2016.08.066

[15] Horseman SV, Buller H, Mullan S and Whay HR. Current welfare problems facing horses in Great Britain as identified by equine stakeholders. PLoS ONE 2016; 11: e0160269 10.1371/journal.pone.0160269 27501387 PMC4976980

[16] Horseman SV, Buller H, Mullan S, Knowles TG, Barr ARS and Whay HR. Equine welfare in England and Wales: Exploration of stakeholders’ understanding. J Appl Anim Welf Sci 2016;14:1–15. 10.1080/10888705.2016.1197776 27414640

[17] Hemsworth LM, Jongman E, and Coleman GJ. Recreational horse welfare: The relationships between recreational horse owner attributes and recreational horse welfare. Appl Anim Behav Sci 2015;165: 1–16.

[18] Schemann K, Firestone SM, Taylor MR, Toribio J-ALML, Ward MP and Dhand NK. Horse owners’/managers’ perceptions about effectiveness of biosecurity measures based on their experiences during the 2007 equine influenza outbreak in Australia. Prev Vet Med 2010;106: 97–107. 10.1016/j.prevetmed.2012.01.013 22326045

[19] Faucitano L, Martelli G, Nannoni E, Widowski T. Fundamentals of Animal Welfare in Meat Animals and Consumer Attitudes to Animal Welfare In:Purslow PP, editor. New Aspects of Meat Quality. Buenos Aires Province, Tandil, Argentina: Woodhead Publishing; 2017 pp 537–568

[20] van der Kolk JH. 34 –Endocrine function during exercise and response to training In: Hinchcliff KW, Kaneps AJ, Geor RJ, editors. Equine Sports Medicine and Surgery, 2nd Edition Philadelphia: Saunders; 2014 pp 769–785.

[21] Waller CC and McLeod MD. A review of designer anabolic steroids in equine sports. Drug Test Anal 2016; 10.1002/dta.2112 27732767

[22] Aven T and Steen R. The concept of ignorance in a risk assessment and risk management context. Reliab Eng Syst Safe 2010;95: 1117–1122.

[23] Faber M, Manstetten R, and Proops JLR. Humankind and the Environment: An Anatomy of Surprise and Ignorance. Environ. Values 1992;1: 217–242

[24] Collins J, Hanlon A, More SJ and Duggan V. The structure and regulation of the Irish equine industries: links to considerations of equine welfare. Ir Vet J 2008; 61: 746–756. 10.1186/2046-0481-61-11-746 21851704 PMC3113872

[25] Michie S, van Stralen MM and West R. The behaviour change wheel: A new method for characterising and designing behaviour change interventions. Implement Sci. 2011; 6: 42 10.1186/1748-5908-6-42 21513547 PMC3096582

