# Supplementary material for: An exploration of industry expert perception of Canadian equine welfare using a modified Delphi technique
Source: PLoS One. 2018 Jul 30;13(7):e0201363. doi: 10.1371/journal.pone.0201363 (PMC6066239; doi:10.1371/journal.pone.0201363)
Supplement: S1 Appendix — (PDF) [file pone.0201363.s001.pdf]

Equine industry expert perception of critical issues in horse welfare in Canada

Q1
CONSENT TO PARTICIPATE IN RESEARCH
Equine industry expert perception of critical issues in horse welfare issues in Canada

You are asked to participate in a research study conducted by *Dr. Katrina Merkies and Cordelie DuBois*, **from the** *Department of Animal Biosciences, Ontario Agricultural College* **at the University of Guelph.** The results of this project will contribute to the fulfillment of a graduate student PhD thesis. (REB # 15DC024)

If you have any questions or concerns about the research, please feel free to contact

Dr. Katrina Merkies: Faculty Supervisor
Associate Professor
Dept. of Animal Biosciences
University of Guelph
Guelph, ON, Canada. N1G 2W1
(519) 824-4120 x54707
kmerkies@uoguelph.ca

Cordelie DuBois, PhD Candidate
University of Guelph
(416) 579-2921
cdubois@uoguelph.ca

PURPOSE OF THE STUDY
This modified Delphi survey-based study is distributed to selected Canadian equine professionals in order to create a consensus on what welfare problems and concerns horses in the Canadian equine industry are facing and how best to assess these problems on-farm through the development of a welfare assessment tool. Furthermore, ranking the importance of these concerns with respect to the individual animal and the Canadian herd provides valuable information for the weighting of sections within the assessment tool. Once major areas of concern for horse welfare are identified, feasible and effective solutions can be developed to improve horse welfare in Canada.

PROCEDURES
If you volunteer to participate in this study, we would ask you to do the following things:
To take part in this survey, you must be 18 years of age and a resident of Canada. This study involves the use of a modified Delphi, which is a structured survey technique that employs the use of several iterative “rounds” of questionnaires to gather and distill information from a panel of experts. In the first round, you will be asked your opinion on the types of welfare issues experienced by horses within the Canadian equine industry and suggestions on how to assess the impact. In the second and third rounds, findings of the first round will be reported and you will be given an opportunity to rank the welfare issues presented by fellow experts. It is

expected that each round should take no more than 40 minutes to complete and your answers are very important to the research! Please take the time to read each question clearly and carefully, and answer honestly and to the best of your ability. If you feel uncomfortable answering any question, you have the option to skip a question. There are no right or wrong answers. Participation in multiple rounds is not mandatory, but is encouraged.

POTENTIAL BENEFITS TO PARTICIPANTS AND/OR TO SOCIETY

The research will provide insights into the perception and understanding of Canadian equine professionals from a variety of different disciplines and fields regarding horse welfare. The results will identify areas of horse welfare concern as well as the relative importance of these areas of concern, which in turn will be form the basis for the creation of a welfare assessment tool. This will ultimately allow for a better understanding of equine welfare issues and potentially inspire others to devise more effective and efficient methods of solving them. Furthermore, the design and scientific testing of a way to measure equine welfare helps to create an international image of the Canadian equine industry as a leader in animal welfare.

CONFIDENTIALITY

Every effort will be made to ensure confidentiality of any identifying information that is obtained in connection with this study.

As this survey involves multiple rounds, email addresses will be collected in order to code the responses. In this way, survey responses from the different rounds can be linked to the same respondent. Once data from all rounds of the survey have been collected, email addresses will be stripped from the data and only the linking code will remain. Data from the survey will be stored on a password-protected computer and only be accessible to the Faculty Supervisor and student researcher. Data will be retained for up to one year after the results of the research are published. Please note that confidentiality cannot be guaranteed while data are in transit over the internet. Participants should clear the browsing history, cache and cookies and log off their computer to help ensure confidentiality, particularly when using a public computer. This can be done in Internet Explorer by selecting Tools – Internet Options – Delete Browsing History (on the General tab).

PARTICIPATION AND WITHDRAWAL

You can choose whether to be in this study or not. If you volunteer to be in this study, you may withdraw at any time before submitting the survey without consequences of any kind by closing your browser. All data entered up to the point of withdrawal will be permanently removed. You may also decline to answer any specific questions you are uncomfortable with and still remain in the study. If you wish to withdraw after submission of your completed survey, you can contact the researchers in writing requesting removal of your data. You are in no way obligated to participate in all rounds of the Delphi questionnaires.

RIGHTS OF RESEARCH PARTICIPANTS

You may withdraw your consent at any time prior to completing the survey and discontinue participation without penalty. You are not waiving any legal claims, rights or remedies because of your participation in this research study. It is recommended that you print this consent form

for your records. This project has been reviewed by the Research Ethics Board for compliance with federal guidelines for research involving human participants. If you have questions regarding your rights as a research participant, contact:

Director, Research Ethics
University of Guelph
437 University Centre
Guelph, ON N1G 2W1

Telephone: (519) 824-4120, ext. 56606
E-mail: sauld@uoguelph.ca
Fax: (519) 821-5236

SIGNATURE OF RESEARCH PARTICIPANT/LEGAL REPRESENTATIVE

By clicking on the box below, you accept that you have read the information provided for the study "*Equine industry expert perception of critical issues in horse welfare issues in Canada*" as described herein. All questions have been answered to your satisfaction, and you agree to participate in this study.

I agree to all terms and conditions described above:

○ Yes (1)

○ No (2)

Q2 **CODE NUMBER:**

____

Q3 Select your gender :

○ Male (1)

○ Female (2)

○ Other/Prefer not to answer (3)

Q4 Select your age category:

- ○ 18-25 years (1)
- ○ 26-35 years (2)
- ○ 36-45 years (3)
- ○ 46-55 years (4)
- ○ 56-65 years (5)
- ○ 65 years or more (6)

Q5
Which province or territory do you reside in?

- ○ Yukon (1)
- ○ Northwest Territories (2)
- ○ Nunavut (3)
- ○ British Columbia (4)
- ○ Alberta (5)
- ○ Saskatchewan (6)
- ○ Manitoba (7)
- ○ Ontario (8)
- ○ Quebec (9)
- ○ New Brunswick (10)
- ○ Newfoundland (11)
- ○ Nova Scotia (12)
- ○ Prince Edward Island (13)

Q6 Please describe your current involvement in the equine industry, listing all or any certifications you have:

____

____

____

____

____

Q7 Approximately how many years have you been involved in the horse industry?

- ○ 0-5 years (1)
- ○ 6-10 years (2)
- ○ 11-20 years (3)
- ○ 21-30 years (4)
- ○ 30 years or more (5)

Q8 Please indicate which equine discipline you are MOST actively participating in (e.g. through competition, lessons, teaching, etc.).

- ◯ English disciplines (English pleasure, eventing, dressage, jumper, hunter, saddleseat) (1)
- ◯ Western disciplines (Western pleasure, cutting, barrel racing, cattle penning, reining, roping, rodeo) (2)
- ◯ Breed competitions (including halter classes) (3)
- ◯ Horse racing (4)
- ◯ Driving (5)
- ◯ Endurance (6)
- ◯ Vaulting and/or Gymkhana (7)
- ◯ Polo (8)
- ◯ Outfitting or trail riding (9)
- ◯ Other, please list: (10) ____

For the following sets of questions, you will first be asked to discuss issues or concerns you believe negatively impact an **individual** horse's well-being (and how you might identify and solve them). Then, on the next page, you will be asked to discuss issues or concerns you believe negatively impact the welfare of horses **in general** at the industry level (and how you might identify and solve them). Please answer the following sets of questions to the best of your ability, drawing on your experience working with and/or in the industry.

Q14 Please list, in your opinion, examples of welfare issues/concerns within the Canadian equine industry that need to be addressed at the **individual horse level**. Welfare issues/concerns can encompass anything you believe reflects or negatively affects a horse's well-being. You are free to list issues/concerns that apply to specific industry sectors and/or to horses in general.

____

____

____

____

Q16 Based on what evidence could you determine if, or to what degree, a horse was experiencing a state of negative well-being, paying particular attention to the issues/concerns you outlined above. (For example, if your welfare concern is ill animals, a way of determining this might be to check the horse's vital signs.)

____

____

____

____

Q18 Please indicate how you personally would best address the issues/concerns you indicated above. (For example, if your welfare concern is ill animals on your farm, the best way to address this may be to contact a veterinarian.)

____

____

____

____

Q21 Please list, in your opinion, examples of welfare issues/concerns within the Canadian equine industry that need to be addressed at the **industry level**. Welfare issues/concerns can encompass anything you believe reflects or negatively affects a horse's well-being. You are free to list issues/concerns that apply to specific industry sectors and/or to horses in general.

____

____

____

____

Q23 Based on what evidence could you determine if, or to what degree, a horse was experiencing a state of negative well-being, paying particular attention to the issues/concerns you outlined above. (For example, if your welfare concern is soring of sport horses, a way of assessing this might be to evaluate the condition of the skin at the fetlock joint.)

____

____

____

____

Q25 Please indicate how you think the issues/concerns you indicated above should be addressed best by industry members. (For example, if your welfare concern is soring of sport horses, the best way to address this may be to increase the number of veterinarian checks at sporting events.)

____

____

____

____

Q10 Please feel free to comment on any aspect concerning the welfare of horses in Canada:

____

____

____

Equine industry expert perception of critical issues in horse welfare in Canada Round 2

Q1
Welcome to round two of the modified-Delphi survey “Equine industry expert perception of critical issues in horse welfare in Canada.”

In the previous round, you were asked to list examples of welfare issues/concerns within the Canadian equine industry that needed to be addressed at the individual horse level and at the industry level. Based on your responses, and the responses of your peers, a collection of welfare issues/concerns and measures/indicators of welfare states was complied.

In this round, you will be asked to rank these issues based on several categories, such as prevalence (in the case of welfare issues). The goal of round two is to move towards a consensus regarding the most critical welfare issues within the equine industry.

As per the consent form you accepted in round one, you may withdraw at any time before submitting the survey without consequences of any kind by closing your browser. If you do not complete round two of the survey you will not be contacted for round three.

Once again, thank you for your time, and if you have any questions or concerns please feel free to contact me or my supervisor.

Cordelie DuBois
PhD Candidate
(416) 579 2921
cdubois@uoguelph.ca

Dr. Katrina Merkies: Faculty Supervisor
Associate Professor
Dept. of Animal Biosciences
University of Guelph
Guelph, ON, Canada. N1G 2W1
(519) 824-4120 x54707
kmerkies@uoguelph.ca

Q12 **CODE NUMBER:**

____

Q2 The following is a list of welfare concerns generated from the first round. These were thought to be best addressed at the level of the **individual horse**. From the list below, please rank them in order in terms of most important (1) to least important (12).

______ Training practices (including excessive use of aids, spike poles for jumpers, overworking horses, working horses at a level beyond their physical abilities, having unreasonable expectations, training horses too young, soring of gaited horses) (1)

______ Drug use (including; lasix in horse racing, joint injections, tail blocking/nerving, misuse of medication, "masking" lameness through painkillers) (2)

______ Too many horses/unregulated breeding (3)

______ Breeding for aesthetic but detrimental traits (4)

______ Horses being denied access to basic physical requirements (e.g. food, water, shelter, turnout) (5)

______ Horses being denied access to important psychological resources (e.g. companionship/social interaction) (6)

______ Improper dietary practices (including overfeeding and obesity, incorrect feeding practices) (7)

______ Lack of proper professional care (veterinarian, dentist, farrier) (8)

______ Lack of daily or attentive monitoring (including taking preventative measures) (9)

______ Lack of knowledge or education (including incorrect information being perpetuated, little value put on evidence-based information, novice owners/owners who don't appreciate time and financial commitment required by horses) (10)

______ Lack of skilled personnel within the industry (including lack of necessary training programs) (11)

______ Lack of long-term planning or end of life care planning (12)

Q3 For each of the following **individual level** welfare issues/concerns, indicate how prevalent you believe they are within the industry on a scale of 0 to 5 with 0 being rare and 5 being prevalent in all industry sectors.

	0 (rare) (1)	1 (2)	2 (3)	3 (4)	4 (5)	5 (all) (6)
Poor training practices (1)	○	○	○	○	○	○
Drug use (2)	○	○	○	○	○	○
Too many horses/unregulated breeding (3)	○	○	○	○	○	○
Breeding for aesthetic but detrimental traits (4)	○	○	○	○	○	○
Horses being denied access to basic physical requirements (e.g. food, water, shelter, turnout) (5)	○	○	○	○	○	○
Horses being denied access to important psychological resources (e.g. companionship/ social interaction) (6)	○	○	○	○	○	○
Improper dietary practices (7)	○	○	○	○	○	○
Lack of proper professional care (veterinarian, dentist, farrier) (8)	○	○	○	○	○	○
Lack of daily or attentive monitoring (including taking preventative measures) (9)	○	○	○	○	○	○
Lack of knowledge or education (10)	○	○	○	○	○	○
Lack of skilled personnel within the industry (11)	○	○	○	○	○	○
Lack of long-term planning or end of life care planning (12)	○	○	○	○	○	○

Q4 For each of the following **individual level** welfare issues/concerns, indicate in the space provided which sector or region you believe these issues are most often found (e.g. the hunter/jumper sector, western Canada).

	Location Most Often Found (1)
Poor training practices (1)	
Drug use (2)	
Too many horses/unregulated breeding (3)	
Breeding for aesthetic but detrimental traits (4)	
Horses being denied access to basic physical requirements (e.g. food, water, shelter, turnout) (5)	
Horses being denied access to important psychological resources (e.g. companionship/social interaction) (6)	
Improper dietary practices (7)	
Lack of proper professional care (veterinarian, dentist, farrier) (8)	
Lack of daily or attentive monitoring (including taking preventative measures) (9)	
Lack of knowledge or education (10)	
Lack of skilled personnel within the industry (11)	
Lack of long-term planning or end of life care planning (12)	

Q9 The following are a list of welfare concerns generated from the first round. These were thought to be best addressed at the **industry** level. From the list below, please rank them in order of what you think is most important (1) to least important (12).
______ Ignorance and lack of knowledge (especially related to horse learning theory and horse behaviour) (1)
______ Overpopulation of horses (including lack of breeding control, unwanted animals) (2)
______ Horse slaughter and horses at feedlots (3)
______ Lack of accountability (including veterinarians not reporting) (4)
______ Lack of standards of care for horses (5)
______ Lack of regulation at the industry level for practices detrimental to welfare (e.g. rules for drug use in competitions) (6)
______ Lack of regulation at the government level supporting equine welfare (7)
______ Lack of knowledge transfer from research to the horse owning community (8)
______ Poor biosecurity practices (9)
______ Perpetuation of outdated or disadvantageous practices (10)
______ Poor public image of the equine industry (11)
______ Lack of long-term planning or end of life care planning (12)

Q13 For each of the following **industry level** welfare issues/concerns, indicate how prevalent you believe they are within the industry on a scale of 0 to 5 with 0 being rare and 5 being prevalent in all industry sectors.

	0 (1)	1 (2)	2 (3)	3 (4)	4 (5)	5 (6)
Ignorance and lack of knowledge (1)	○	○	○	○	○	○
Overpopulation of horses (2)	○	○	○	○	○	○
Horse slaughter and horses at feedlots (3)	○	○	○	○	○	○
Lack of accountability (4)	○	○	○	○	○	○
Lack of standards of care for horses (5)	○	○	○	○	○	○
Lack of regulation at the industry level for practices detrimental to welfare (6)	○	○	○	○	○	○
Lack of regulation at the government level supporting equine welfare (7)	○	○	○	○	○	○
Lack of knowledge transfer from research to the horse owning community (8)	○	○	○	○	○	○
Poor biosecurity practices (9)	○	○	○	○	○	○
Perpetuation of outdated or disadvantageous practices (10)	○	○	○	○	○	○
Poor public image of the equine industry (11)	○	○	○	○	○	○
Lack of long-term planning or end of life care planning (12)	○	○	○	○	○	○

Q14 For each of the following **industry level** welfare issues/concerns, indicate in the space provided which sector or region you believe these issues are most often found (e.g. the hunter/jumper sector, western Canada).

	Location Most Often Found (1)
Ignorance and lack of knowledge (1)	
Overpopulation of horses (2)	
Horse slaughter and horses at feedlots (3)	
Lack of accountability (4)	
Lack of standards of care for horses (5)	
Lack of regulation at the industry level for practices detrimental to welfare (6)	
Lack of regulation at the government level supporting equine welfare (7)	
Lack of knowledge transfer from research to the horse owning community (8)	
Poor biosecurity practices (9)	
Perpetuation of outdated or disadvantageous practices (10)	
Poor public image of the equine industry (11)	
Lack of long-term planning or end of life care planning (12)	

Q8 Given the list of welfare issues/concerns provided by you and your peers, list possible reasons or motivations as to why these issues/concerns appear in the industry.

Equine industry expert perception of critical issues in horse welfare in Canada Round 3

Q2
Welcome to round three of the modified-Delphi survey "Equine industry expert perception of critical issues in horse welfare in Canada."

In the previous round, you were asked to rank the welfare issues listed in round one, as well as assess multiple welfare measures based on several categories, such as prevalence (in the case of welfare issues). You were also asked to provide a list of possible reasons or motivators as to why these issues/concerns appear in the industry. Based on your responses, and the responses of your peers, a collection of motivators was complied, as well as a list of potential solutions to welfare issues/concerns (as collected from the first round).

In this round, you will be asked to rank the listed motivators with respect to how much they contribute to equine welfare concerns within the industry. You will also be asked to rank the effectiveness of the potential solutions with respect to addressing equine welfare concerns on an individual and then on an industry level.

The goals of round three are 1) to move towards a consensus regarding the best ways to address welfare concerns within the industry and 2) to determine which motivators are the primary drivers behind poor welfare situations.

As per the consent form you accepted in round one, you may withdraw at any time before submitting the survey without consequences of any kind by closing your browser.

Once again, thank you for your time, and if you have any questions or concerns please feel free to contact me or my supervisor.

Cordelie DuBois
PhD Candidate
(416) 579 2921
cdubois@uoguelph.ca

Dr. Katrina Merkies: Faculty Supervisor
Associate Professor
Dept. of Animal Biosciences
University of Guelph
Guelph, ON, Canada. N1G 2W1
(519) 824-4120 x54707
kmerkies@uoguelph.ca

Q4 **CODE NUMBER:**

____

Q8

Below is a list of potential methods that Round 1 respondents indicated could be used to address animal welfare concerns. Please rank them (in your opinion) in order of most effective (1) to least effective (14) with respect to addressing equine welfare concerns on an INDIVIDUAL horse level. To rank them, please drag each item into the order you wish them to appear.

______ Allow horses to have more access to physical requirements (e.g. food, water, shelter, turnout) (1)

______ Utilize veterinary equipment to determine if procedures are necessary (e.g. make use of ultrasound equipment before joint injections are performed) (2)

______ Contact/report cases to a regulation body (e.g. Society for the Prevention of Cruelty to Animals, Ontario Racing Commission/Association) (3)

______ Make proper horse care the primary goal (as opposed to winning, for example) (4)

______ Industry stake holder involvement (e.g. initiate educational sessions for horse owners) (5)

______ Regulate breeding (6)

______ Strengthen and enforce animal welfare legislation (7)

______ Education (more centralized, increased awareness, more reliable sources, pre-purchase knowledge) (8)

______ Better understanding of equine behaviour and behavioural cues (9)

______ Change rules and regulations issued by equine associations (e.g. redefine competition judging standards to better reflect natural horse behaviours) (10)

______ Daily checks of animals and their housing systems (e.g. fence checks) (11)

______ Change perception of practices (e.g. what constitutes a good trainer) (12)

______ Better communication between equine professionals (veterinarians, farriers, nutritionists, dentists, etc.) and owners (13)

______ Consistent routine care (e.g. hoof trimming, dental exams) (14)

Q9 Below is a list of potential methods that Round 1 respondents indicated could be used to address animal welfare concerns. Please rank them (in your opinion) in order of most effective (1) to least effective (20) with respect to addressing equine welfare concerns on an INDUSTRY level. To rank them, please drag each item into the order you wish them to appear.

______ Records of sales and transfers of ownership of horses (1)

______ Collect industry data to serve in the creation of benchmarks for acceptable standards (e.g. career duration of competitive horses) (2)

______ Increased control of drug usage in the competition sector (includes harsher penalties, mandatory intermittent drug testing, increased accountability) (3)

______ Cooperation within the industry to work towards common goals (includes creating a united front when approaching the government for assistance or support) (4)

______ Require horse owners to be licensed/registered before owning animals (to ensure proper knowledge regarding horse care) (5)

______ Better understanding of equine behaviour and learning theory (6)

______ Restriction or banning of live horses exported from Canada for the purposes of slaughter (7)

______ Education for all people dealing with horses (e.g. owners, farriers, feed companies) - includes continuing education priorities for organization members, targeted public education, responsible horse ownership, teaching through welfare advocates, education on horse needs (8)

______ Increased number of officials at competition events (includes required veterinary checks before, during, and after competitions for all animals) (9)

______ Regular inspections of facilities (with special attention paid to training methods) (10)

______ Decreased incentives for utilizing young horses in competition (includes age restrictions) (11)

______ Development of evidence-based tools in order to better assess equine welfare (12)

______ Alter the way horses are judged (focusing more on conformation and sound movement rather than imposed aesthetics) (13)

______ Restriction or banning of live horses imported to Canada for the purposes of slaughter (14)

______ Mandatory welfare training for officials who oversee horse welfare (includes educating policy makers - especially in the government- regarding horse needs) (15)

______ Equine associations acting as leaders and advocates of good practice (16)

______ Increased control of horse slaughter (includes better awareness, harsher penalties for fraudulent dealings, stricter drug testing, industry-recognized identification system) (17)

______ The creation of a universal definition of equine welfare (18)

______ Increased provincial and/or federal regulation (19)

______ Changes in legislation for competition horses (increased safety of horses competing) (20)

Q6
Below is a list of potential motivators that Round 2 respondents indicated could be responsible for or contribute to compromised welfare. Please rank them in order from most important (1) to least important (14) with respect to how much you believe they contribute to compromised equine welfare within the industry. To rank them, please drag each item into the order you wish them to appear.

______ Lack of animal welfare legislation (insufficient and lenient penalties, not properly used) (1)
______ Human convenience (e.g. providing concentrate feed in discrete meals) (2)
______ Limited equine research (3)
______ Lack of resources involved in investigation and prosecution (e.g. in equine abuse/neglect cases) (4)
______ Financial gain (e.g. associated with competition, desire to win) (5)
______ Anthropomorphism (attributing human emotions to animals) (6)
______ Ignorance/lack of education (at the government level) (7)
______ Tradition (8)
______ Willful neglect and abuse (9)
______ Ignorance/lack of education (at the owner level) - includes the lack of knowledge that horses are a lifetime commitment (10)
______ Financial difficulties (lack of resources) (11)
______ Lack of leadership in the equine community (12)
______ Horses as disposable commodities (13)
______ Lack of access to professionals (e.g. in remote areas) (14)
