# Supplementary material for: An exploration of industry expert perception of Canadian equine welfare using a modified Delphi technique
Source: PLoS One. 2018 Jul 30;13(7):e0201363. doi: 10.1371/journal.pone.0201363 (PMC6066239; doi:10.1371/journal.pone.0201363)
Supplement: S1 Fig — (PDF) [file pone.0201363.s003.pdf]

Round 1	Round 2	Round 3
Participant demographics (e.g. age, location, industry role)	*Round 1 review*	*Round 2 review*
Brainstorm examples of welfare issues/concerns (industry and individual level)	Rank welfare issues/concerns	Rank effectiveness of ways to address welfare concerns
Brainstorm ways of addressing welfare concerns (industry and individual horse level)	Indicate perceived prevalence of issues/concerns	Rank importance of motivator contribution to welfare issues/concerns
	Indicate where issues/concerns were most often found	
	Brainstorm potential motivators behind welfare issues/concerns	
